# Performance Characterization of an *xy*-Stage Applied to Micrometric Laser Direct Writing Lithography

**DOI:** 10.3390/s17020278

**Published:** 2017-01-31

**Authors:** Juan Jaramillo, Artur Zarzycki, July Galeano, Patrick Sandoz

**Affiliations:** 1 Grupo de Investigación Electromagnetismo Aplicado, línea Microingeniería, Universidad EAFIT, Medellín 050022, Colombia; jjaram44@eafit.edu.co; 2 Grupo de Investigación en Automática, Electrónica y Ciencias Computacionales, Línea Sistemas de Control y Robótica, Instituto Tecnológico Metropolitano, ITM, Medellín 050013, Colombia; 3 Grupo de Investigación en Materiales Avanzados y Energía MatyEr, Línea Biomateriales y Electromedicina, Instituto Tecnológico Metropolitano, ITM, Medellín 050013, Colombia; julygaleano@itm.edu.co; 4 Department of Applied Mechanics, FEMTO-ST Institute, University Bourgogne Franche-Comté, UFC/CNRS/ENSMM/UTBM, Besançon 25000, France; psandoz@univ-fcomte.fr

**Keywords:** laser direct writing, photolithography, microfabrication, G-Code, high-resolution positioning, *xy*-stage

## Abstract

This article concerns the characterization of the stability and performance of a motorized stage used in laser direct writing lithography. The system was built from commercial components and commanded by G-code. Measurements use a pseudo-periodic-pattern (PPP) observed by a camera and image processing is based on Fourier transform and phase measurement methods. The results report that the built system has a stability against vibrations determined by peak-valley deviations of 65 nm and 26 nm in the *x* and *y* directions, respectively, with a standard deviation of 10 nm in both directions. When the *xy*-stage is in movement, it works with a resolution of 0.36 µm, which is an acceptable value for most of research and development (R and D) microtechnology developments in which the typical feature size used is in the micrometer range.

## 1. Introduction

In microfabrication and micro-electronics industries, a widely used method for the realization of microstructured features consists in realizing the desired circuit on a binary mask and then transferring these features on a series of substrates by a photolithography process. This approach allows the realization of low-cost, multilayered structures covering extended surfaces, substrate diameters up to 300 mm, and that can be produced in large series within very short time frames. The processes involved require, however, very expensive technological facilities than can be justified/affordable only for continuous mass production. However, when low-series or single units are needed, especially for research and development (R and D) purposes, the realization of a photolithography mask becomes an intermediary step that presents significant drawbacks in regard to cost and delays. For this reason, several mask-less photolithography techniques have been developed recently.

The mask-less lithography technique is more flexible than conventional mask lithography. The former has possibilities to fabricate arbitrary shapes with ultrahigh-resolution and with a minimum feature size as small as a few nanometers [1,2]. Additionally, mask-less lithography promises to make chip personalization easy and to be particularly cost effective for low-volume chip designs. This approach may also allow home-processing with technological secrecy benefits. Mask-less lithography technologies are based on different techniques, such as electron beam lithography [3,4], focused ion beam lithography [5], scanning probe lithography [6], electrohydrodynamic direct-writing lithographic [7], and laser direct writing (LDW) lithography [8,9,10]. The LDW technique is very popular due to its ease of use, flexibility, and cost effectiveness, especially for micrometer-sized R and D processing.

Ready-to-use laser direct writing devices are commercially available but they are very specialized and, thus, very expensive. Meanwhile, research groups and small laboratories need cheaper and more versatile solutions which can be easily adjusted to the need of the hour. This includes home-made devices composed from commercial components which answer very well to the needs.

In comparison with typical photolithography, the LDW method presents some advantages. It allows modifying, correcting, or designing new patterns without unnecessary delay (the new pattern can be directly transferred to the substrate, achieves a 3D or gray-tone pattern), by controlling the optical system, and permits work on substrates with non-standard-sizes. It is important to note that well-assembled LDW devices can also be used to produce photolithography masks.

The LDW photolithography setups are usually based on a motorized stage, an optical system equipped with a UV laser, and a table which holds a substrate covered by photoresist [11,12]. The desired pattern on the substrate is obtained by properly coupling the stage displacements with the on/off control of the laser. The motorized stage can be used in three different configurations: (a) the table with the substrate is fixed and the laser moves according to the predesigned pattern; (b) the laser is fixed and the table moves; and (c) both the table and the laser move. Whatever the configuration, the system built must present sufficient resolution/accuracy performances to fulfill the requirements of the microfabrication task. The characterization of those different components is, therefore, a necessary preliminary task to the assembly of a direct lithography machine [13].

For the motorized stage, two approaches can be used: a stacked *xy*-stage or a coplanar *xy*-stage. Both types of stages have cumulative errors due to their mechanical assembly, those errors being smaller in the coplanar stage than in the stacked one. However, the coupling of the two motors in the same plane, as in the case of a coplanar stage, makes the command of this type of stage more difficult [14,15,16]. Moreover, those stages are less versatile than the stacked ones. In this way, the stacked stages result in an appropriate option for low-cost direct writing machines.

The cumulative errors due to the mechanical assembly of different stages induce a decrease of the overall repeatability, precision, and resolution performances. Those errors constitute a major source of problems for accurate alignment and position feedback as it is nowadays needed in the industry of micro- and nanofabrication.

For accurate alignment procedures, techniques based on image processing of fiducial marks have been proposed. Lee et al. [14] propose a technique for alignment of a coplanar *xy*-stage. In this work, an image of a cross was selected as a fiducial mark. A floating-reference point-based image alignment method is presented, reporting alignment accuracy of ±1 µm and ±5 arc-sec.

In the paper presented by Lin et al. [15], the authors propose a visual method based on neural network for precise mask alignment of an *xy-* (coplanar) stage by means of a fiducial mark. The authors report maximum rotational and positioning errors of 0.013° and 17.0 µm.

In both papers, the image processing techniques present error values larger than those that can be achieved with positioning techniques based on phase analysis by Fourier transform [17,18,19].

Other authors [20] report on a system composed of linear precision stages (*x*, *y*) stacked with piezoelectric motors (which offer nanometric precision), and a laser interferometer system for precise measurement of position. This measuring system is used for position feedback for displacement control.

From the *xy*-stage point of view, the use of piezo systems is an expensive solution providing limited displacement ranges compared to step motors.

Regarding displacement control, although interferometric systems are highly-accurate position-measuring systems, their main drawback is related to the loss of the position when the laser beam is interrupted. In such cases the measurement system is down.

Concerning low-cost techniques reported in the literature, Liu et al. [21] present a rapid prototyping technique for microfluidics by using galvanometer mirrors. The technique achieves pattern resolution of up to 25 µm. Greer et al. [12] report a laser lithography system from one customized CNC machine. The *xy* accuracy of the machine is around 50 µm. In both mentioned studies, no method for precise alignment or position feedback is presented.

In this paper, the authors report on the characterization of the mechanical behavior of a stacked *xy*-stage of a laser lithography machine developed from commercial components. The configuration of the machine is with fix laser and movable substrate holder table. The characterization was carried out by means of a highly-accurate absolute visual method based on a pseudo-periodic pattern (PPP) and Fourier transform. This study corresponds to a characterization step preliminary to the use of the mentioned visual method in actual photolithography alignment and positioning.

## 2. Materials and Methods

### 2.1. Presentation of the Laser Direct Writing Machine

#### 2.1.1. Hardware

To ensure *xy* movements of the platform, two crossly-stacked NLS4 linear stages (Newmark System, Inc., Rancho Sta Marg, CA, USA) were used. Both stages are equipped with stepper motors guaranteeing 150 mm (6″) travel with 30 nm resolution. Additionally, on the top, a rotary (*θ*) stage was stacked to allow an easy alignment of the substrate with respect to the axes of the *xy*-stage when necessary at the substrate installation step. This rotation element is made of an RT-3 stage (Newmark System, Inc., Rancho Sta Marg, CA, USA) ensuring angular displacement with 0.29 arc-sec resolution and ended with sample vacuum holder. This supplementary stage is not indispensable to LDW and its performances are, therefore, not discussed in this paper.

The optical system uses a 405 nm laser diode as UV light source (BML-405 Lasermate Group, Inc., Walnut, CA, USA). The laser beam passes through a mechanical shutter and is then deflected by a dichroic mirror (Thorlabs DMLP490R, long pass dichroic mirror, 490 nm cutoff) that directs the beam onto the sample via a microscope lens. The lens is mounted onto a movable stage equipped with a micrometric screw, thus allowing focus adjustment when necessary, especially on samples with a variable thickness. We used a 20× magnification lens with a long working distance of 12 mm and numerical aperture (N.A) of 0.4 (Olympus LMPLFLN-BD, Olympus Corporation, Shinjuku, Tokyo, Japan) permitting the placement of the sample with ease. The microscope objective can be then used in two ways: (1) to focus the UV light beam onto the substrate with the desired spot size; and (2) to form the image of the PPP on the camera with visible light. A monochromatic CMOS camera (DCC3240N from Thorlabs, Newton, NJ, USA) was mounted on the optical axis in the direction of dichroic mirror transmission. The camera helps in controlling beam focalization and in acquiring images for measurements. The camera has a resolution of 1280 × 1024 pixels, and has a pixel size of 5.3 µm^2^. Illumination of the PPP is performed by means of an additional white light source that is transmitted by the dichroic mirror.

**Figure 1:**
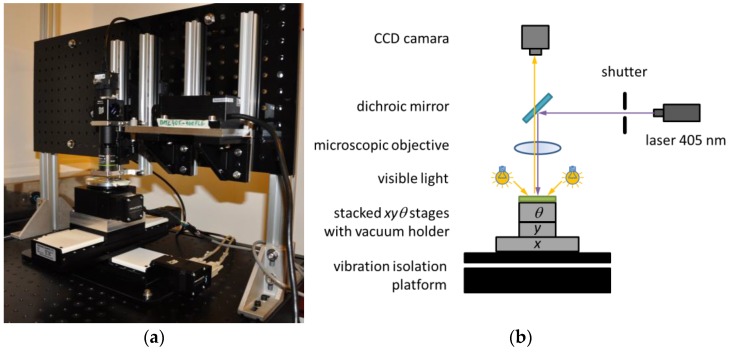
LDW setup developed from commercial components: (**a**) photo of the system; and (**b**) schematic diagram of the system.

The *xyθ*-stage, as well as the optical system, were placed on a vibration isolation platform (250BM-1 Minus K^®^ Tech. Inc., Inglewood, CA, USA). The load capacity of the platform is in range 82–122 kg. The system described is presented in Figure 1. We also note that with the magnification and N.A. used, the size of the focused UV laser beam varies slowly versus the *z* position of the microscope objective, thus making the system robust against micrometer-sized disturbances along the *z*-direction.

#### 2.1.2. Software

Although the *xy*-stage can be programmed with the motor manufacturer’s software, it can be convenient to program it by G-code, which is the most widely used language for numerical control machines, e.g., mills, lathes, drills, laser cutters, etc. G-code is a set of commands that define coordinates in a working space within which the tool must move, the trajectory that the tool must follow, and how fast the tool must move with respect to a workpiece. Therefore, while the commands are generated, the workpiece geometry, desired geometry, as well machining data, such as tool type and size, spindle speed, tool trajectory, etc., must be defined.

**Figure 2:**
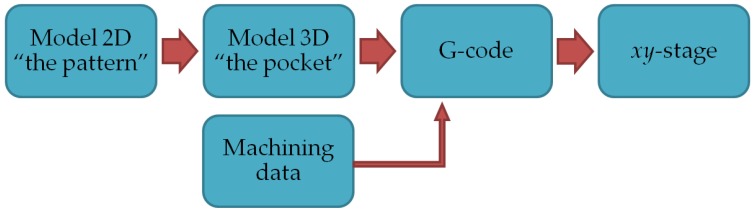
Conversion scheme of CAD files to G-code for monitoring the *xy*-stage displacements.

Microfabrication designs are available mainly in CAD files with extensions such as .gds, .dxf, .stl, and so on. Since the *xy*-stage presented in this article is being controlled by G-code, a conversion from CAD design to G-code has to be carried out. The latter involves a 3D model as schemed in Figure 2.

The conversion of the CAD model to G-code can be explained through the case of an elementary shape represented by a simple square with 100 µm edge length. In this case, several machining parameters have to be defined, among which some are important for the LDW application, whereas other are irrelevant to the need. The first important parameter is the type of machining. In the case presented in this article, milling was chosen as the machining type. Indeed, the whole area of the desired shape has to be exposed to the UV light; therefore, the whole surface has to be scanned as in milling. Another important parameter is the type of tool. In this case, an end mill with a diameter of 20 µm was chosen. In our application, this important parameter stands for the laser beam diameter.

Milling acts in both vertical and horizontal directions and thus converts the substrate surface into a 3D shape of which depth was defined to be of 50 µm, despite this parameter having no significance with respect to our photolithography application. We then obtain the 3D pocket shape.

Another important parameter is the feed rate, set to 50 µm/s, which defines the velocity with which the tool advances along the workpiece. In LDW, this parameter determines the exposure time.

**Figure 3:**
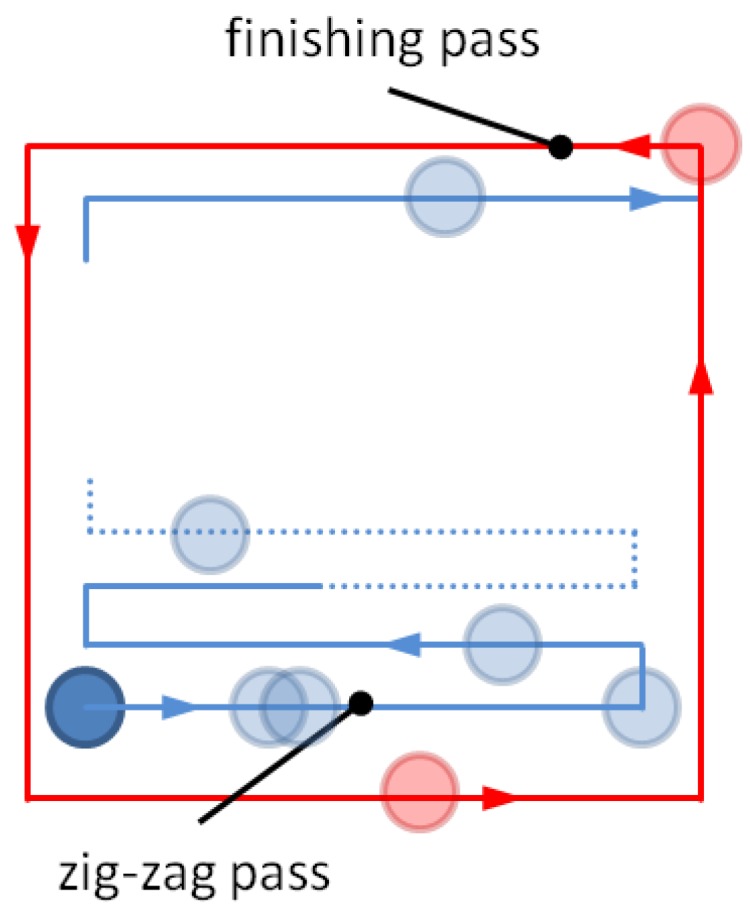
Zig-zag tool-path approach scheme.

Other important parameters are: the tool trajectory or tool path, and step-over. The latter was set to 2 µm and indicates the distance between centerlines of adjacent tool paths. There are several types of tool path approaches in pocket milling. However, they can be divided into two main groups [22]: contour-parallel and direction-parallel. The first one is generated by successive offsets of the input profile whereas the second one uses line segments that are parallel to an initially-selected reference line. The approach chosen to generate the tool path is of importance because of influencing parameters such as: machining time, cutting forces, length of the tool path, and surface roughness [23]. That issue is also important in LDW photolithography and is a subject of further work. For the present paper, only one approach was selected. Since in the proceeding experiments the pocket was in a square shape, the direction-parallel approach and particularly zig-zag tool-path was selected, as shown in Figure 3. In that approach the pocket is tooled in two steps: in the first the tool follows the zig-zag path (roughing tooling—after the zig-zag pass the pocket size is usually smaller than designed), and in the second one the tool follows the pocket’s perimeter (finishing tooling). This approach was selected due to the reduced machining time (the material is removed in both forward and backward tool’s movement), and thanks to the trajectory’s geometry, parameters, such as repeatability, accuracy, and resolution, can be read out with ease.

### 2.2. Fourier-Based Vision Method for Direct Writing Machine Characterization

#### 2.2.1. Pseudo Periodic Pattern (PPP)

**Figure 4:**
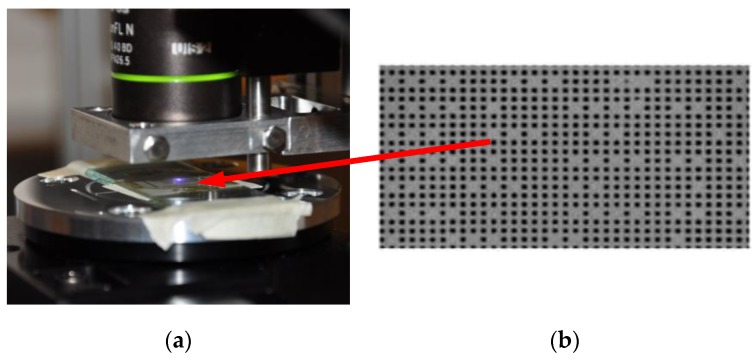
(**a**) Pseudo-periodic pattern placed on the stacked stages; and (**b**) example of a zoomed area of a recorded image (the full field of view (FOV) of the image is 408 µm × 508 µm).

The in-plane position measurement system developed aims to make lateral resolution and range of allowed displacement independent of each other. To this end, we use a PPP placed over the *xyθ*-stage (Figure 4a). The physical extension of those PPP is of one square centimeter with a specific absolute position encoding that allows to identify the location of any tiny zone under observation among the whole PPP surface. In practice, the PPP is made of a periodic distribution of dots altered by a small proportion of missing dots that perform the encryption of the absolute position with respect to the whole pattern (Figure 4b) [17,18]. In this design, the periodic frame is used for the obtaining of a high resolution thanks to interpolation based on phase measurements, whereas the missing dots provide a complementary, coarse position information. The PPP period is 4 µm and provides a physical size reference directly available in images without calibration whereas the point size is about 2 µm.

#### 2.2.2. Processing of PPP Images

The processing of PPP images involves two steps. The first one is based on Fourier transform and allows highly-accurate relative position retrieval in both *x*- and *y*-directions, as well as the in-plane orientation (*β*). This step involves phase computations in which one PPP period corresponds to a 2*π* phase shift. The orientation β is analog to the angle θ associated to the rotation stage but its origin refers to the PPP design rather than to the rotation device. The second step is based on the binary decoding of PPP images and provides the coarse, but absolute, x,y position. The results of those two steps are combined to obtain the final (x,y,β) absolute-high-accuracy position of the area under observation on the microscope.

The Fourier transform of PPP images results in a spectrum formed by lobes. Those lobes are representative of the spatial frequencies of the PPP and contain the information about the vertical and horizontal position of the PPP within the image. Then, single lobes are filtered out and, by means of inverse Fourier transform, the phases relative to the associated directions of the PPP are retrieved. Phase maps are obtained in a wrapped way due to periodicity of the inverse tangent function involved in this calculation. Thus, phase maps have to be compensated with 2π constants by means of an operation known as phase unwrapping [24,25]. The unwrapped phase planes can then be fitted by a first degree equation since the PPP periodicity leads to a linear phase distribution (φ) with respect to the image pixel frame. This is obtained by means of least square fitting, notably for the two perpendicular elementary directions $\varphi_{V}$ and $\varphi_{H}$ of the PPP, as shown by Equations (1) and (2) below [18]:

(1) $$\varphi_{V}\left(u,v\right)=A_{V}\cdot u+B_{V}\cdot v+C_{V}$$

(2) $$\varphi_{H}\left(u,v\right)=A_{H}\cdot u+B_{H}\cdot v+C_{H}$$

where:

(3) $$A_{V}=\frac{2\pi }{pM}\cos\left(\beta \right)$$

(4) $$A_{H}=\frac{2\pi }{pM}\sin\left(\beta \right)$$

(5) $$B_{V}=\frac{2\pi }{pM}\sin\left(\beta \right)$$

(6) $$B_{H}=\frac{2\pi }{pM}\cos\left(\beta \right)$$

(7) $$C_{V}=2k\pi +\Delta \varphi_{V}$$

(8) $$C_{H}=2k\pi +\Delta \varphi_{H}$$

In these equations p is the physical period of the fringes, M is the magnification, and $\Delta \varphi_{V}$ and $\Delta \varphi_{H}$ are phase shifts encoding the position of the two directions of the PPP with respect to the image pixel frame. $C_{H}$ and $C_{V}$ parameters are obtained with an ambiguity of a multiple of 2π because phase unwrapping is a process that depends on the starting point. (u,v) are the coordinates in pixels of the computed phase map images, *β* and is the angle between the PPP orientation, and the horizontal axis of the image pixel frame and can be retrieved by:

(9) $$\beta =arctan\left(\frac{A_{H}}{B_{H}}\right)$$

or

(10) $$\beta =arctan\left(\frac{B_{V}}{A_{V}}\right)$$

Underscript letters H and V can be seen as referring to the horizontal and vertical directions of the PPP, respectively. However, these directions are not related to the recorded image pixel frame and can, therefore, correspond to any orientation in the recorded images, depending on the actual orientation of the PPP with respect to the fixed camera.

In the second step, the distribution of missing points is decrypted in order to localize the current view with respect to the whole PPP. This step involves the identification of the missing points that is performed by means of a local contrast evaluation [18]. The binary values allow the removal of the ambiguity of the constants $C_{H}$ and $C_{V}$ from Equations (1) and (2). In this way, the highly-accurate but relative position obtained by Fourier processing is converted into absolute and high-accurate position once $C_{H}$ and $C_{V}$ constants have been adjusted to fit with the coarse position provided by this binary code decryption.

Full details about PPP image processing, decoding and performances can be found in [17,18].

## 3. Results

As presented in Section 2.2, a PPP was used together with an image processing technique based on Fourier transform in order to estimate the stability, repeatability, and resolution of the implemented *xy*-stage for LDW lithography. Additonally, the effects of the milling parameters on the stage’s behavior were evaluated. The measurements were carried out in two conditions of the stage: (a) in a static state; and (b) in a dynamic state when doing a pocket figure. The results are presented in the following sections.

### 3.1. Vibrations

**Figure 5:**
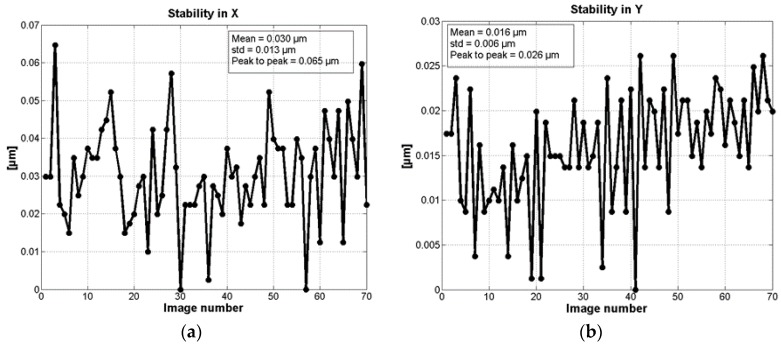
Measurement of the stability of the system in static mode. (**a**) Stability in the *x*-direction; and (**b**) stability in the *y*-direction.

With the anti-vibratory stage switched on (active) and the *xy*-stages static, in-plane measurements were obtained. In this case, images of the PPP were acquired through the monochrome camera and the 20× objective, with an exposition time of 7 ms and a frame rate of 33 fps. A total of 70 frames were acquired. The system was operating in ambient conditions without intentional sources of vibrations. The results are presented in Figure 5.

The results in the Figure 5 present a peak-valley deviations of 65 nm and 26 nm in the *x*- and *y*-directions, respectively, while the standard deviations are 10 nm in both directions with respect to the corresponding mean values.

### 3.2. Path Programming: Pocket Figures and the Effects of the Milling Parameters on the Stage’s Behavior

A pocket figure was programmed and its trajectory’s geometry was performed by the *xy*-stage. As described in Section 2.1.2, the pocket figure used for the following analysis, corresponds to one square of size 100 × 100 µm. It was created first in CAD software and then translated into G-code (cf. Section 2.1.2).

Images of the PPP were acquired when the *xy*-stage was following the programmed pocket figure. The camera was set to a rate of 20 fps. With the pocket milling parameter settings of this work, a total of 32 frames per line were acquired. In this way, each frame corresponded to one sampled point of the pocket scanning.

**Figure 6:**
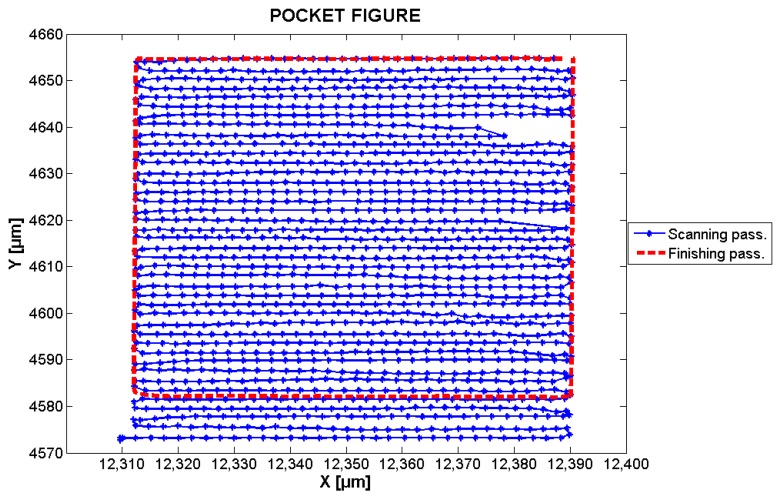
Reconstructed x,y trajectory followed by the stage. There is a lack of data in the trajectory in the line *Y* = 4640 µm and 4620 µm. This is due to imperfections in the acquired images (irregular hard disk drive access is suspected as a possible cause).

**Figure 7:**
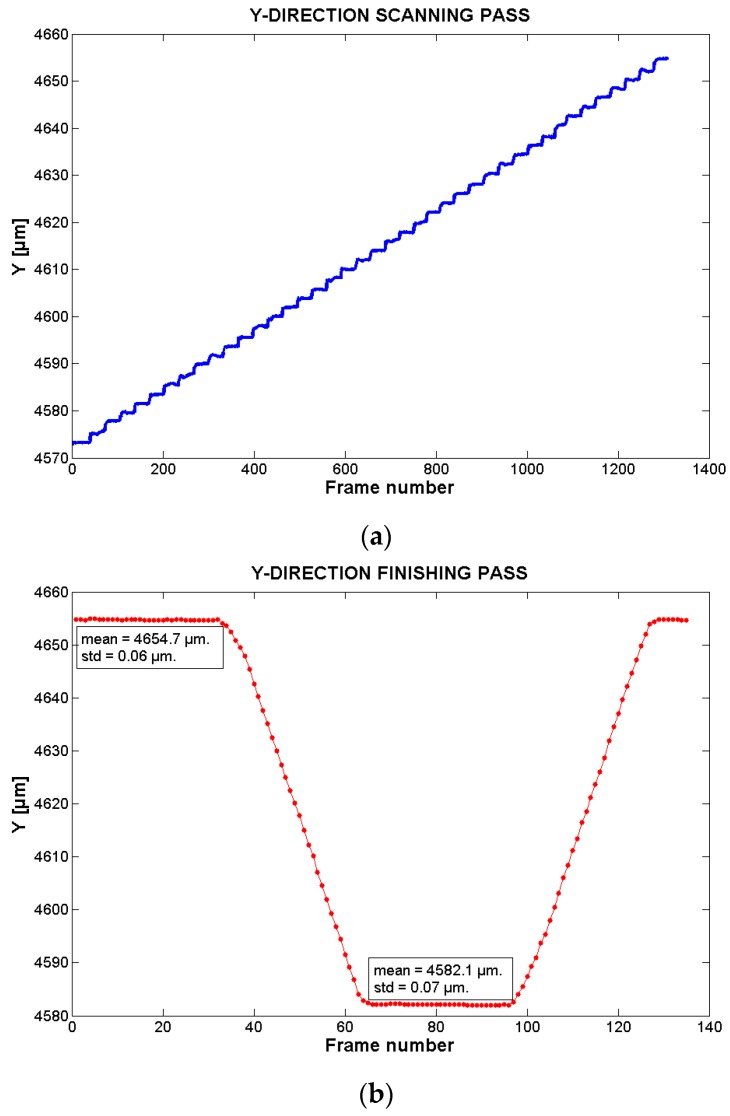
Trajectory followed by the stage in *y*-direction during: (**a**) the scanning pass (zig-zag pass); and (**b**) the finishing pass.

**Figure 8:**
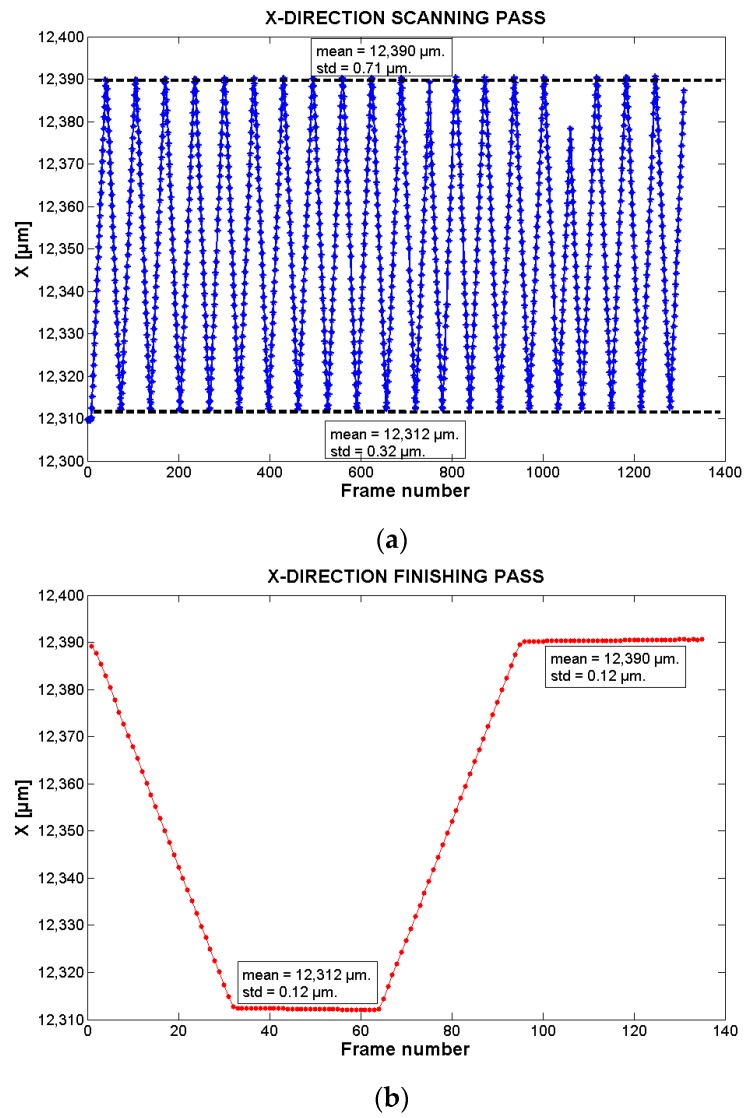
Trajectory followed by the stage in *x*-direction during: (**a**) the scanning pass (zig-zag pass); and (**b**) the finishing pass. The mean and STD values are calculated from the minimum and maximum values of the trajectories. In (**a**) there is a lack of data in the trajectory between frame number 1000 and 1200. This is due to imperfection in the acquired images.

After the processing of each frame through the Fourier-based method, the *xy* absolute highly-accurate position of the corresponding sampled point is known. The results of the programmed pocket are presented in Figure 6, Figure 7 and Figure 8.

Figure 6 presents the trajectory followed by the stage in both *x-* and *y*-directions. The blue-dotted lines correspond to the scanning pass (zig-zag path), while the red-dashed line corresponds to the finishing pass. In both cases, the marks correspond to sampled point coordinates obtained from the processing of PPP images.

Figure 7 and Figure 8 present the trajectory followed by the stage in the *y*- and *x*-directions, respectively.

Table 1 presents the mean, standard deviation (STD), and step values of the *y*-direction during the scanning pass. The scanning pass is divided in 41 steps, each one corresponding to the *y*-position of the scanning lines of the pocket figure. The STD values of the table represent how much the *y*-positions of the sampled points per step differ from the step’s mean y-position. The STD average value is 0.12 µm.

The STD results show that although the system is in movement (due to the scanning in the *x*-direction), the *y*-direction is stable enough to maintain the set step *y*-value. From this STD value, the resolution of the *y*-direction can be estimated to be 0.36 µm (3*σ*), when the stage is in movement. This result can also be extended to the *x*-direction stage.

The step values are the difference between the current step mean value and the previous one. In average, the step values correspond to 2 µm, which is congruent with the *step-over* parameter set during the G-code generation. Additionally, this average value is a good indicator of the system capabilities to follow trajectories with small steps of the order of few micrometers.

Figure 8a corresponds to the *x*-direction of the scanning pass. From this graphic, it is possible to analyze the system repeatability along this direction. In the pocket figure, the stage has to follow a trajectory that, in the *x*-direction, corresponds to go from a starting value to an ending one, and then to return back. In the figure, the mean value of the minimum and maximum points per trajectory (extreme values) is 12,312 µm and 12,390 µm respectively. The STD in the maximum and minimum points are 0.12 µm in both cases. This is an indicator of the system repeatability along this direction, which is estimated to be of 0.36 µm (3*σ*).

Figure 7b and Figure 8b present the trajectory followed by the system in the *y*-direction and *x*-direction respectively during the finishing pass. In the figures, it is possible to observe that at the beginning, the system changes its *x* position from 12,390 µm to 12,312 µm while the *y*-value is kept in a steady state at a value of around 4654.7 µm (frames 1–32). Then, the system stays/maintains static in the *x*-direction while the *y*-direction changes from 4654.7 µm to 4582.1 µm in a linear way (frames 34–63). Then, the *y*-direction stays static in this last value while the *x*-direction moves from the minimum value to the maximum one (frames 65–96). Once at the maximum, the *x*-direction stays static, while the *y*-direction moves from its minimum to its maximum (frames 96–135).

## 4. Discussion

The difference between the mean value of the minimum and maximum points per scanning trajectory in the *x*-direction and *y*-directions are 78.0 µm and 81.5 µm, respectively. This corresponds to the width of the pocket figure created by the scanning pass. Compared to the set pocket size (100 µm), it is possible to observe that there is a difference of 22.0 µm and 18.5 µm. Bearing in mind the distance that should be between the starting and ending points per direction in the trajectory (80 µm due the milling tool size—Section 2.1.2), there is an absolute difference of only 2 µm and 1.5 µm. This differences can be explained by the milling programming leaving some material to remove when doing the finishing pass.

In the case of the finishing pass, the difference between the mean value of the minimum and maximum points in the *x*-direction and *y*-direction are 78.0 µm and 72.6 µm. For the *x*-direction, it is similar to the results of the scanning trajectory. However, by comparing the behavior of the system in the *y*-direction in both the zig-zag and finishing passes, it is possible to observe that the maximum values are similar while the minimum values are not. The difference is that while the zig-zag pass is moving according to the set pocket trajectory, the finishing pass has a difference of 7.4 µm in the minimum *y*-scanning value. This error can be due to the stages bidirectional repeatability which, according to the stages’ manufacturer, is in the range of 10 µm. This is an error that is proper/inherent of the *xy*-stages but that could be compensated by the visual method in a future work.

## 5. Conclusions and Future Work

In this paper, the authors report on the performance characterization of a stacked *xy*-stage, which is part of a laser direct writing photolithography system made from commercial elements. The system is commanded by G-code which is a language widely used in numerical control machines. The characterization was carried out by means of pseudo-periodic pattern (PPP) and a visual method based on Fourier transforms.

The results report that the built system has a stability against vibrations determined by peak-valley deviations of 65 nm and 26 nm in *x* and *y* directions, respectively, with a standard deviation of 10 nm in both directions over a time lapse of about 2 s. When the *xy*-stage is in movement, it works with a resolution of 0.36 µm, which is an acceptable value for most of R and D microtechnology developments in which the typical feature size used is in the micrometer range.

Additionally, the results made it possible to analyze the effects of the G-code commands over the photolithography patterning. Since the original design is done in the form of a CAD-CAM file, a conversion to G-code has to be performed. This conversion implies appropriately setting a series of parameters corresponding to milling machining.

The performed characterization shows that the technique of PPP images processed by a Fourier-based method is a potential tool for photolithography alignment and positioning procedures. Thanks to the high-accurate-absolute measurements that are obtained with this technique, it would be possible to compensate positioning errors due to stages’ repeatability and resolution, as well as external misalignments or vibrations. With the proposed PPP technique, a resolution of around 11 nm and accuracy better than 5 nm can be achieved [26,27] with insensitivity to data interruption during measurements, contrary to interferometer systems. The system can easily resume its work from the current position, which can be identified at any moment in an absolute manner. This means that a system based on PPP can be used either for the full DWL positioning feedback or as a fiducial mark for wafer alignment.

Future work concerns the analysis of the optical system, as well as the performances of the LDW device in photolithography practice.

## Figures and Tables

**Table 1:** Mean, STD, and step values of the *y*-direction scanning pass.

Step Number	Mean Value (µm)	STD (µm)	Step Value (µm)
1	4573.22	0.02	0.00
2	4575.31	0.25	2.09
3	4577.84	0.03	2.53
4	4579.59	0.11	1.76
5	4581.48	0.03	1.89
6	4583.43	0.09	1.95
7	4585.55	0.17	2.12
8	4587.48	0.30	1.93
9	4589.94	0.06	2.45
10	4591.65	0.16	1.71
11	4593.63	0.07	1.98
12	4595.53	0.10	1.90
13	4597.90	0.20	2.37
14	4599.85	0.33	1.94
15	4601.95	0.10	2.10
16	4603.81	0.13	1.86
17	4605.72	0.10	1.92
18	4608.02	0.32	2.30
19	4610.02	0.09	2.00
20	4612.00	0.10	1.98
21	4614.00	0.02	2.00
22	4616.10	0.19	2.10
23	4617.86	0.06	1.76
24	4619.90	0.15	2.04
25	4622.16	0.02	2.26
26	4624.08	0.04	1.91
27	4626.12	0.06	2.04
28	4628.05	0.09	1.93
29	4630.25	0.16	2.20
30	4632.39	0.10	2.13
31	4634.39	0.08	2.01
32	4636.35	0.12	1.95
33	4638.13	0.12	1.79
34	4640.65	0.11	2.51
35	4642.59	0.09	1.94
36	4644.47	0.10	1.88
37	4646.56	0.09	2.08
38	4648.41	0.15	1.86
39	4650.27	0.13	1.85
40	4652.25	0.39	1.98
41	4654.73	0.04	2.48
